# Unusual coexistence of oral lymphoepithelial cyst and benign migratory glossitis

**DOI:** 10.1016/S1808-8694(15)30798-9

**Published:** 2015-10-19

**Authors:** Karuza Maria Alves Pereira, Cassiano Francisco Weege Nonaka, Pedro Paulo de Andrade Santos, Ana Myriam Costa de Medeiros, Hébel Cavalcanti Galvão

**Affiliations:** 1 Master's degree in oral pathology; doctoral student in the oral pathology graduate program; 2 Master's degree in oral pathology; doctoral student in the oral pathology graduate program; 3 Dental prosthetic care specialist; master's degree student in the oral pathology graduate program; 4 Doctorate in oral pathology; professor of the Oral Diagnosis Discipline; 5 Doctorate in oral pathology; professor of the oral pathology graduate program. Universidade Federal do Rio Grande do Norte

**Keywords:** lymphoepithelial cyst, oral lymphoepithelial cyst, benign migratory glossitis

## INTRODUCTION

The lymphoepithelial cyst is an uncommon lesion in the mouth; it manifests as a discrete asymptomatic whitish-yellow elevation, generally located on the floor of the mouth.^1^^,^^2^ Benign migratory glossitis may be characterized by erythematous patches with whitish margins across the surface of the tongue, with periods of exacerbation and remission that confer the typical migratory aspect of this entity.^3^ At the time of this publication, this is the first case reporting a coexistence of oral lymphoepithelial cyst and benign migratory glossitis.

## CASE REPORT

A female patient aged 46 years visited the Serviço de Diagnóstico Oral complaining of episodic burning sensation on the tongue, with period in which the condition disappeared; this had lasted for several years. The intraoral examination showed multiple painful erythematous patches with white contours, located on the dorsum of the tongue; the diagnosis was benign migratory glossitis. The patient was prescribed supportive measures that consisted of corticosteroids (0.1 mg/ml dexamethasone chloridrate) for the relief of symptoms.

**Figure 1:**
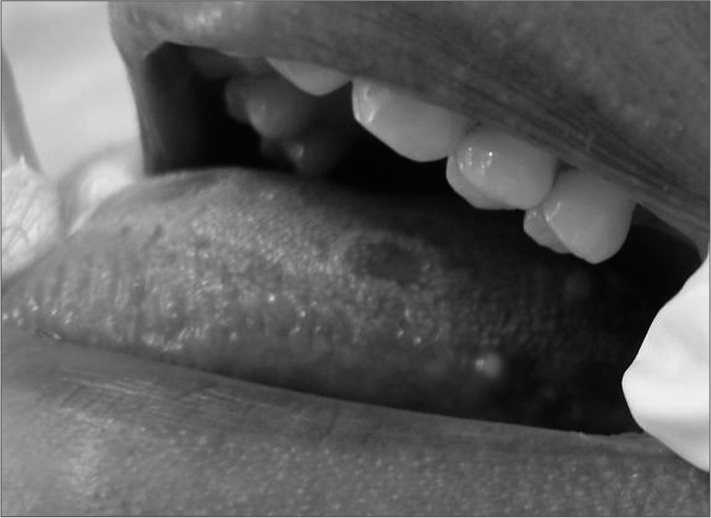
Photography showing erythematous lesions with a discrete white snaky border. A yellow papule is seen on the posterolateral surface of the tongue.

Careful inspection revealed a painless yellowish papule in the posterolateral portion of the tongue; its diameter was about 5 mm, and the patient had not perceived this lesion until then (Fig. 1). An excision biopsy was done and the material was sent to pathology (Serviço de Anatomia Patológica). The microscope examination showed a cavity lined with parakeratinized stratified squamous epithelium with a flat epithelial-connective tissue interface; the capsule consisted of a fibrous connective tissue with protruding lymphoid tissue and formation of germinative centers. The diagnosis was lymphoepithelial cyst.

No recurrence of the oral lymphoepithelial cyst was observed after a one-year follow-up period. There were three symptomatic recurrences of the benign migratory glossitis; all were treated palliatively with corticosteroids (0.1mg/ml dexamethasone chrolidrate).

## DISCUSSION

Oral lymphoepithelial cysts are rare. Many of these cysts are located on the floor of the mouth (65.3 %); about 13.7% of cases are located on the posterolateral portion of the tongue. Oral lymphoepithelial cysts are asymptomatic and small, rarely having a diameter over 15 mm.^1^^,^^2^ In our case, the presence of a small lymphoepithelial cyst in an uncommon site underlines the importance of a careful clinical examination.

There have been few reports on the coexistence of oral lymphoepithelial cysts and epidermoid cysts in the same anatomical site.^4^ An analysis of cases of benign migratory glossitis shows that this condition has been associated with a number of medical local or systemic conditions.^5^ Until the present date, however, no case with an oral lymphoepithelial cyst and benign migratory glossitis in the same patient had been reported.

The pathogenesis of lymphoepithelial cysts is not understood; possible causes that have been suggested are local trauma and obstructed tonsillary crypts.^2^^,^^4^ The etiopathogenesis of benign migratory glossitis is also a matter of debate in the literature; this condition has not been associated with trauma or obstructive events.^5^ The coexistence of an oral lymphoepithelial cyst and benign migratory glossitis, as seen in the case, is probably an uncommon association between diseases with different etiologies.

The treatment of oral lymphoepithelial cysts consists of conservative surgical removal; recurrences are rare.^2^^,^^4^ In the case above, there has been no recurrence one year after excision biopsy. Three recurring symptomatic episodes of benign migratory glossitis have been observed in this case; all were treated with supportive corticosteroid therapy, as described in the literature for other such cases.^6^

## COMMENTS

Until the present date, this is the first case report of an oral lymphoepithelial cyst coexisting with benign migratory glossitis. Although the origin of these lesions is unclear, their etiopathogenesis is distinct. Thus, the coexistence of an oral lymphoepithelial cyst and benign migratory glossitis is probably an uncommon association between these diseases.

Furthermore, lymphoepithelial cysts are small lesions. This case report underlines the importance of a careful examination of the mouth, beyond the patient's main complaint.
